# Genetic Diversity of Potential Drug Resistance Markers in *Plasmodium vivax* Isolates from Panama, Mesoamerica

**DOI:** 10.3390/pathogens14030231

**Published:** 2025-02-27

**Authors:** Vanessa Vásquez, Ana María Santamaría, Dianik Moreno, Fergie Ruíz, Chystrie A. Rigg, Luis F. Chaves, José E. Calzada

**Affiliations:** 1 Departamento de Investigación en Parasitología, Instituto Conmemorativo Gorgas de Estudios de la Salud, Panamá 0816-02593, Panama; vvasquez@gorgas.gob.pa (V.V.); asantamaria@gorgas.gob.pa (A.M.S.); chrigg@gorgas.gob.pa (C.A.R.); 2 Laboratorio Central de Referencia en Salud Publica, Instituto Conmemorativo Gorgas de Estudios de la Salud, Panamá 0816-02593, Panama; dmoreno@gorgas.gob.pa (D.M.); fruiz@gorgas.gob.pa (F.R.); 3 Department of Environmental and Occupational Health, School of Public Health, Indiana University, Bloomington, IN 47408, USA; 4 Department of Geography, Indiana University, Bloomington, IN 47405, USA; 5 Facultad de Medicina Veterinaria, Universidad de Panamá, Panamá 0816-3366, Panama

**Keywords:** malaria, *Plasmodium vivax*, drug resistance genes, genetic diversity, Panama, Mesoamerica

## Abstract

This study evaluated the genetic diversity and potential drug resistance markers in *Plasmodium vivax* isolates from Panama, a country in Mesoamerica, aiming to eliminate local malaria transmission. We analyzed 70 *P. vivax* samples collected between 2004 and 2020 from endemic regions in Eastern and Western Panama, as well as imported cases. Four drug resistance genes (*pvcrt-o*, *pvmdr1*, *pvdhfr*, and *pvdhps*) were sequenced and analyzed. Our findings reveal low genetic diversity in *P. vivax* populations from Western Panama, indicating clonal expansion, while Eastern Panama exhibits higher diversity, influenced by higher transmission rates and imported cases. No mutations were detected in *pvcrt-o*, and the prevalence of *pvmdr1* mutations (Y976F and F1076L) linked to chloroquine was observed at low frequencies, primarily in imported samples. In *pvdhfr*, antifolate-resistant mutations S117N and S58R were detected in 14.3% of samples, predominantly from Eastern Panama near the Colombian border. Phylogenetic and haplotype network analyses highlighted distinct genetic clustering, supporting the influence of imported cases on local parasite diversity. These results provide a baseline for the molecular surveillance of *P. vivax* in Panama and emphasize the need for the continued monitoring of genetic diversity and drug resistance to guide regional malaria elimination efforts, particularly in areas with high cross-border migration.

## 1. Introduction

**Figure 1:**
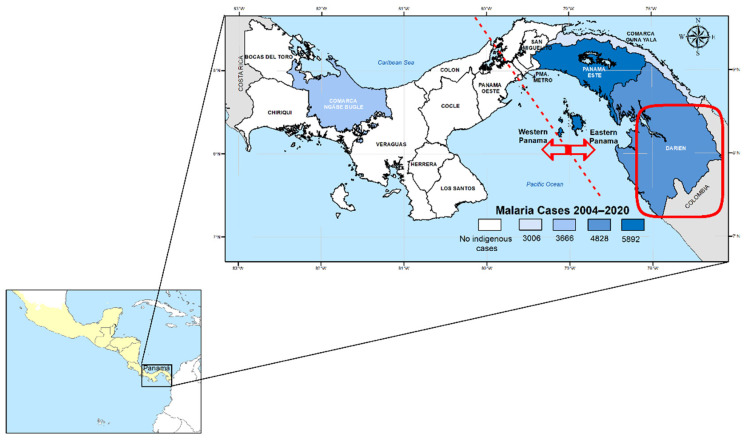
A map of Panama showing health regions with active *Plasmodium vivax* transmission based on the cumulative number of cases between 2004 and 2020. The dashed lines in the map indicate the Panama Canal pathway that artificially divides the country into Eastern and Western Panama. The red square represents the location of the Darien Gap consisting of a road-less swath of swampland and rainforest within Panama’s Darién Province and the northern portion of Colombia’s Chocó Department. The Darien Gap is a corridor used by migrants from around the globe who intend to reach the United States or Canada. The lower inset map shows the location of Panama within the Mesoamerican region, colored in yellow.

Panama is a malaria-endemic country situated in a strategic position at the southeastern end of Mesoamerica (Figure 1). Despite active engagement in global (E-2020) and regional initiatives (REMEI) aimed at interrupting local malaria transmission by 2020 and 2022, respectively [1,2], Panama has fallen short of achieving these goals [3]. More recently, the country recommitted to eliminating malaria by 2025 in a renewed E2025 initiative sponsored by the World Health Organization (WHO) [4,5]. Since the launch of the National Malaria Elimination Plan (NMEP) in 2018 [6], malaria transmission in the country has shown a concerning upward trend [7]. This is evidenced by a dramatic rise in the number of indigenous cases, escalating from 684 in 2018 to 11,057 in 2023—a staggering increase of over 1600% [3]. Alarmingly, this trend continued into 2024, with a further 31% increase in malaria cases compared to the previous year, with a rate of increase of 1.31 times compared to 2023 (14,476 cases vs. 11,053 cases).

Compounding this epidemiological scenario, the NMEP in Panama, already debilitated by resource reallocation towards the COVID-19 response, faces additional challenges. These include the health threats posed by climate change and, notably, the mass human migration crossing the Darien Gap in Panama aiming to reach the United States or Canada [3].

In recent decades, the burden of malaria in Panama has been predominantly concentrated among highly mobile indigenous populations inhabiting remote areas, characterized by limited access to healthcare services [7]. Recent observations indicate that *Plasmodium falciparum* transmission is circulating at low levels in several localities in the eastern region of the country. However, *P. vivax* is by far the predominant species across all endemic regions, accounting for more than 98% of the total indigenous malaria cases officially reported between 2010 and 2020 [7]. This *P. vivax* dominance trend has persisted in recent years. Therefore, to effectively achieve malaria elimination in Panama, gaining a better understanding of the biology of this species and the development of *P. vivax*-specific strategies is a priority in the national malaria research agenda towards elimination.

Regarding malaria vectors, several species have been documented in Panama, with *Anopheles albimanus* and *An. punctimacula* being the most common and widely distributed across the country. Nevertheless, there are other anophelines species that could be implicated in malaria transmission in the different endemic regions of the country. For instance, *An. (Nys.) darlingi* has recently been described in specific regions from Eastern Panama [8]. Indoor residual spraying with alternating different insecticides, including organophosphates, pyrethroids, and neonicotinoids, has been the main vector control intervention in Panama. In recent years, the NMEP has also implemented long-lasting insecticidal nets in highly endemic communities [9].

In addition to the numerous and distinctive challenges impeding the control of *P. vivax*, such as the presence of latent hypnozoite stages and early gametocytogenesis, there is a growing concern regarding the potential spread of drug-resistant vivax malaria within the region. While the resistance of *P. vivax* to chloroquine (CQ) has already reached concerning levels in several Southeast Asian countries [10,11], in the Americas, few countries have described well-documented *P. vivax* resistance, primarily nations within the Amazon basin [11]. Currently, within the Mesoamerican subregion, which includes southeastern Mexico and all of the Central American countries, there have been no reports of chloroquine-resistant *P. vivax* malaria [11]. In fact, except for Panama, even indigenous strains of *P. falciparum* circulating in this subregion continue to exhibit sensitivity to chloroquine.

In Panama, as in all malaria-endemic countries in the WHO region of the Americas, CQ is the first-line treatment for *P. vivax* blood-stage parasitemia, coupled with primaquine (CQ + PQ) to eliminate persistent liver-stage (hypnozoite) infections [3,10,12]. While this combination remains highly effective for treating *P. vivax* infections, recent anecdotal reports have indicated a growing number of malaria recurrences, particularly in endemic areas of Eastern Panama. However, the underlying causes of these suspected treatment failures have not been thoroughly investigated. There is also concern regarding the increasing number of imported *P. vivax* cases linked to mass migration from regions where *P. vivax* has developed resistance to chloroquine [7]. This situation raises alarms about the potential spread of drug-resistant parasites within Panama and Mesoamerica as a region [13].

Monitoring *P. vivax* drug resistance relies primarily on therapeutic efficacy studies, which are resource-intensive, costly, and particularly challenging to conduct in remote areas with highly mobile populations [11]. Molecular methods provide a partial solution to these challenges as they can be performed retrospectively, allowing for high-throughput screening and frequent sampling of parasite populations. Various mutations (nucleotide substitutions, indels, and copy number variation) have been identified in *P. vivax* orthologs of genes associated with *P. falciparum* resistance [11]. Some degree of genetic association has been observed between mutations in *pvcrt*-o and *pvmdr1* with chloroquine resistance, as well as between mutations in *pvdhfr* and *pvdhps* with antifolate resistance [11,14]. Although these associations are not as robust or well characterized as those observed in *P. falciparum*, these molecular markers have been proposed as tools for monitoring *P. vivax* drug resistance and for conducting retrospective studies to track molecular changes over time [15,16].

Likely because it has not yet been perceived as a significant issue impacting *P. vivax* control in the Mesoamerican region, there have been very few studies evaluating the potential resistance of this malaria parasite species to chloroquine and other antimalarial drugs. However, the emerging epidemiological scenario, characterized by a significant increase in malaria cases, including the growing detection of imported cases from regions where resistance has been reported, highlights the urgent need to conduct such studies in the region. To address this knowledge gap, the objective of the present study was to assess the frequency of mutations and the genetic diversity of *pvcrt*-o, *pvmdr1*, *pvdhfr*, and *pvdhps* genes in both autochthonous and imported *P. vivax* parasites circulating in endemic regions of the country. The regional implications of these molecular findings for the control of *P. vivax* malaria to attain the shared elimination goal are discussed.

## 2. Materials and Methods

### 2.1. Study Site and Blood Sample Collection

Malaria samples and epidemiological data analyzed in this study were collected by personnel from the National Malaria Control Programme (NMCP) as part of routine surveillance conducted across all endemic areas in Panama (Figure 1). Clinical blood samples were obtained via finger prick from patients with malaria, including both autochthonous and imported cases, between 2004 and 2020.

### 2.2. Ethical Statement

A retrospective molecular analysis of the samples and the protocols used for molecular surveillance was approved by the Departamento de Control de Vectores of the Ministry of Health (No. 147/DCV/DGSP and No. 374/DCV/ICG) and by the Comité de Bioética de la Investigación del Instituto Conmemorativo Gorgas de Estudios de la Salud (No. 468/CNBI/ICGES/06, No. 413/CNBI/ICGES/12 and No. 314/CBI/ICGES/24). The confidentiality of the study subjects with malaria was protected, and individual data were not shared.

### 2.3. Plasmodium vivax Diagnosis

Blood samples were utilized for the preparation of thick blood smears for routine diagnostic purposes and were also spotted onto filter paper for subsequent molecular analysis. Genomic DNA was extracted from the dried blood spots using the QIAamp DNA Mini Kit (Qiagen, Hilden, Germany) following the manufacturer’s protocol for dried blood spots. To confirm *P. vivax* in smear-positive samples, a nested PCR was performed targeting the small subunit ribosomal RNA (ssrRNA) genes following a modified methodology from the protocol by Snounou et al. [17]. Specifically, as *P. vivax* and *P. falciparum* are the only malaria species circulating in Panama, the second PCR reaction included species-specific primers for these two parasites exclusively.

### 2.4. Amplifications and Sequencing of Potential P. vivax Drug Resistance Genes

Samples diagnosed as single *P. vivax* infections by microscopy and nested PCR were genotyped by amplifying and sequencing the following drug resistance markers: *pvcrt*-o, *pvmdr1*, *pvdhfr*, and *pvdhps*.

### 2.5. PCR Amplifications and Sequence Alignment

Amplification reactions were performed in a final volume of 50 μL containing 25 μL of Go Taq Green Master Mix 2X (Promega, Madison, WI, USA), 1 μM of each primer, and 5 μL of DNA purified from blood samples.

A *pvcrt*-o gene fragment of approximately 1137 bp was amplified using the following primers: F3: 5′-ATCCCGTCATCCGCCTCACT-3′ and R3:3′-AGTTTCCCTCTACACCCG-5′ [18]. PCR was performed under the following conditions: 94 °C for 5 min and 40 cycles at 94 °C for 30 s, 56 °C for 30 s, and 72 °C for 2 min, followed by a final extension at 72 °C for 10 min.

A *pvmdr1* gene fragment of approximately 564 bp was amplified using the following primers: Pvmdr1-4F 5′-CCCTCTACATCTTAGTCA TCG-3′ and Pvmdr1-4R 5′-TGGTCT GGACAAGTATCTAAAA-3′ [19]. PCR was performed under the following conditions: 95 °C for 5 min and 40 cycles at 94 °C for 30 s, 56 °C for 30 s, and 72 °C for 2 min, followed by a final extension at 72 °C for 10 min.

For the *pvdhfr* gene, a 700 bp fragment was amplified with primers F: 5′-ATGGAGGACCTTTCAGATGTATT-3′ and R: 5′-CCACCTTGCTGTAAACCAAAAAGTCCAGAG-3′ [20]. For *pvdhps*, a nested PCR approach was performed following the protocol previously described in [20]. Briefly, a 1300 bp fragment was amplified in the second amplification with primers F2: 5′-GGTTTATTTGTCGATCCTGTG-3′ and R2: 5′-GAGATTACCCTAAGGTTGATGTATC-3′. The following PCR cycling conditions were used for *pvdhfr* and *pvdhps* amplifications: 94 °C for 10 min; 45 cycles of 94 °C for 5 s, 58 °C for 1 min, 72 °C for 2 min, and a final extension at 72 °C for 10 min.

PCR products were confirmed via electrophoresis in a 1.5% agarose gel with a 0.5X TBE buffer. The amplicons were then purified using a Qiagen DNA purification kit (Qiagen, Hilden Germany). To ensure suitability for sequencing, the quality and concentration of the DNA were evaluated measuring the absorbance at 260 and 280 nm with a NanoDrop spectrophotometer. Purified products were then directly sequenced in both directions with the Sanger method using the same primers described for amplification and an ABI Prism 3500 XL130 sequencer (Applied Biosystems, Foster City, CA, USA). The sequences for each gene were visually checked for the detection of any missing or ambiguous (heterozygous) sites when two peaks overlapped in a chromatogram. Nucleotide sequences were edited and aligned with Sequencher 4.1.4 and Molecular Evolutionary Genetics Analysis (MEGA) 11 software (Pennsylvania State University, Center, PA, USA).

Nucleotide sequences from this study were submitted and registered in GenBank under the following accession numbers: pvmdr1 (OL763561-OL763624 and PP663644-PP663649), pvdhfr (OL763625-OL763688 and PP657422-PP657427), pvdhps (OL763689-OL763752 and PP662634-PP662639), and pvcrt (OL763753-OL763816 and PP662640-PP662645) (Table S1).

### 2.6. Phylogenetic and Genetic Diversity Analyses

To explore genetic diversity and conduct a population structure analysis, the sequences of the four drug resistance markers (*pvcrt*-o, *pvmdr1*, *pvdhfr*, and *pvdhps*) were concatenated into a single sequence for each sample of 2738 bp. Multilocus sequences were aligned using MEGA 7.0 software, and a phylogenetic tree was constructed by the maximum likelihood method with 1000 bootstrap replicates [21,22]. The following reference sequences from different malaria-endemic regions were obtained from GenBank and included in the phylogenetic analysis:

Sal-1: AAKM01000014.1, AAKM01000011.1, AAKM01000006.1, and AAKM0100001.1;

CMB-1: LQRK01000782.1, LQRK01002034.1, LQRK01002914, and LQRK01000000;

North Korea: AFNJ01000025.1, AFNJ01001278.1, AFNJ01001955.1, and AFNJ01000612.1;

India VII: AFBK01000017.1, AFBK01001286.1, AFBK01000659.1, and AFBK01001976.1;

Brazil I: AFMK01000017.1, AFMK01001011.1, AFMK01000563.1, and AFMK01001407.1;

Mauritania I: AFN101000015.1, AFN10100079.4, AFN101000377.1, and AFN101001277.

The PopART program version 1.7 [23] and Median Joining algorithm were used to construct a haplotype network of Panamanian *P. vivax* populations based on the four genes concatenated. To compute genetic diversity indices, local samples were grouped as follows by their geographic origin: Western Panama, Eastern Panama, and imported cases. The latter was inferred based on travel history. DnaSP Version 6.12.03 [24] was used to estimate the number of haplotypes (H), haplotype diversity (Hd), segregating sites (S), nucleotide diversity (π), total number of mutations (Eta), the mean number of pairwise differences (k), and neutrality tests (Fu’s Fs statistics).

## 3. Results

The study population initially comprised 150 *P. vivax* PCR-confirmed blood samples collected in Panama between 2004 and 2020 as part of routine malaria surveillance. Of these samples, successful genotyping of the four combined drug resistance genes was achieved in 70 cases. The patients originated from all recognized endemic regions in the country, including the western areas (Veraguas and Bocas del Toro) and the eastern regions (Panamá Este, Guna Yala, and Darién) of the Panama Canal (Figure 1).

Twelve samples were collected from patients diagnosed in health facilities located in the non-endemic metropolitan region of Panama (Panama Metro); however, the precise geographical origin of these infections could not always be determined based on the patients’ declared travel histories. Additionally, seven samples were obtained from patients who reported traveling to malaria-endemic countries: four from South America and three from Asia.

### 3.1. Genetic Polymorphisms in Drug Resistance Genes

All sequences were analyzed using the *P. vivax* Sal1 strain as the reference. In the amplified *pvcrt*-o gene fragment (1137 bp), no mutations were identified in the coding regions of this gene. However, a point mutation in an intronic region was identified at position 185 nt (C/T). This mutation was observed in imported cases (PO03, PO05, PO06, PO28, and PO37) and in samples from communities in Eastern Panama, including Panama Metro (PM03, PM11, PM19, and PM21), Guna Yala (KY149, KY164, and KY166) and Darien (DA61, DA64, and DA63) (Tables S1 and S2). Additionally, a mutation at position 578 nt (T/C) was detected in samples from eastern Darien (DA61, DA64, and DA63).

**Figure 2:**
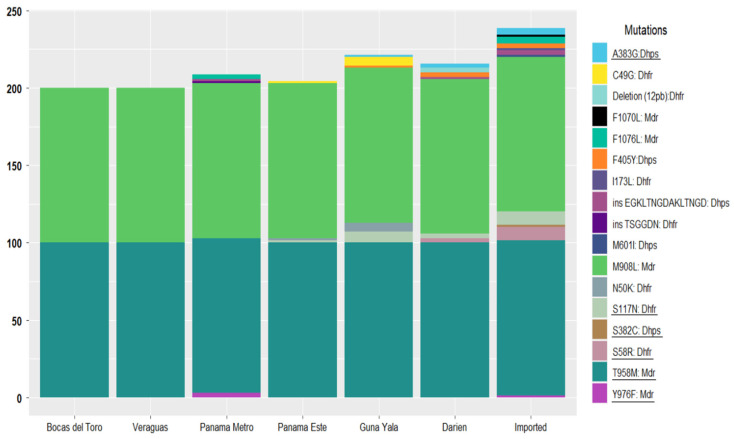
The mutation frequencies of *pvcrt*-o, *pvmdr1*, *pvdhfr*, and *pvdhps* genes in *P. vivax* isolates from malaria-endemic regions in Panama and assumed imported cases based on patients’ travel history. Detected mutations are represented by different colors. The mutations associated with drug resistance are underlined. The Y-axis represents the % of samples with a mutation for each region. This % is defined as the proportion (×100) of samples from each region where a mutation was detected. The percentages are presented in an additive manner so that for each mutation, the % is the difference between the highest and lowest values for each gene in the bar representing each studied region. Because of the additive presentation, the values on the Y-axis go over 100%.

In the *pvmdr1* gene, five nonsynonymous mutations were detected: M908L, T958M, Y976F, F1070L, and F1076L (Figure 2 and Table S2). The M908L and T958M mutations were fixed across all local and imported samples. Conversely, Y976F, a mutation associated with chloroquine resistance, was detected in three samples (3/70; 4.3%), including one imported from Iquitos, Peru, and two from the non-endemic metropolitan region, with unclear infection origins. The F1070L mutation was observed in a single imported sample from the Brazilian Amazon (1/70; 1.4%), while F1076L was present in five samples (5/70; 7.1%), three of which were imported (India, Peru, and Venezuela), and two of which were from non-endemic metropolitan areas.

In the *pvdhfr* gene, seven mutations were identified, including five nonsynonymous point mutations (C49G, N50K, S58R, S117N, and I173L) and one 12 bp deletion (Figure 2). Notably, the C49G and N50K mutations were exclusively present in the same five local samples from the eastern region of Guna Yala. The S58R mutation, which is associated with pyrimethamine resistance, was detected in eight samples (11.4%), six of which, according to their travel history, were imported cases from China, India, Peru, Brazil, and Venezuela, while two were collected from Comarca Emberá Wounaan in Darién near the border of Colombia. These two samples were the only ones in the study population that also harbored the 12 bp deletion. The S117N mutation, linked to pyrimethamine resistance, was found in 20% of the samples (14/70), distributed across seven imported cases and seven samples from patients residing in eastern communities along the northward migration route. The I173L mutation was exclusively observed in an imported case from Mumbai, India. Tandem repeat variations were also investigated in the *pvdhfr* gene. Most samples (69/70) presented allelic variant type C (deduced amino acid sequence: GGDNTS GGDNAD). Only one sample collected from a community in the non-endemic metropolitan region held the type B variant (GGDNTS GGDNTH GGDNAD). This sample also shared the Y976F mutation, known to be associated with chloroquine resistance (Figure 2 and Table S2).

Five nonsynonymous mutations were identified in the *pvdhps* gene (S382C, A383G, F405Y, M601I, and the EGKLTNGDAKLTNGD insertion), predominantly in imported cases (Figure 2). The S382C and M601I mutations, associated with sulfadoxine resistance, were detected in single samples from Venezuela and India, respectively. The nonsynonymous F405Y mutation was the most frequently observed in the *pvdhps* gene, appearing in 7.1% of the samples, with four cases originating from Eastern Panama and one imported from China. The EGKLTNGDAKLTNGD insertion was identified in two samples imported from South America and two locals from Eastern Panama.

### 3.2. Phylogenetic Tree and Haplotype Network Results

**Figure 3:**
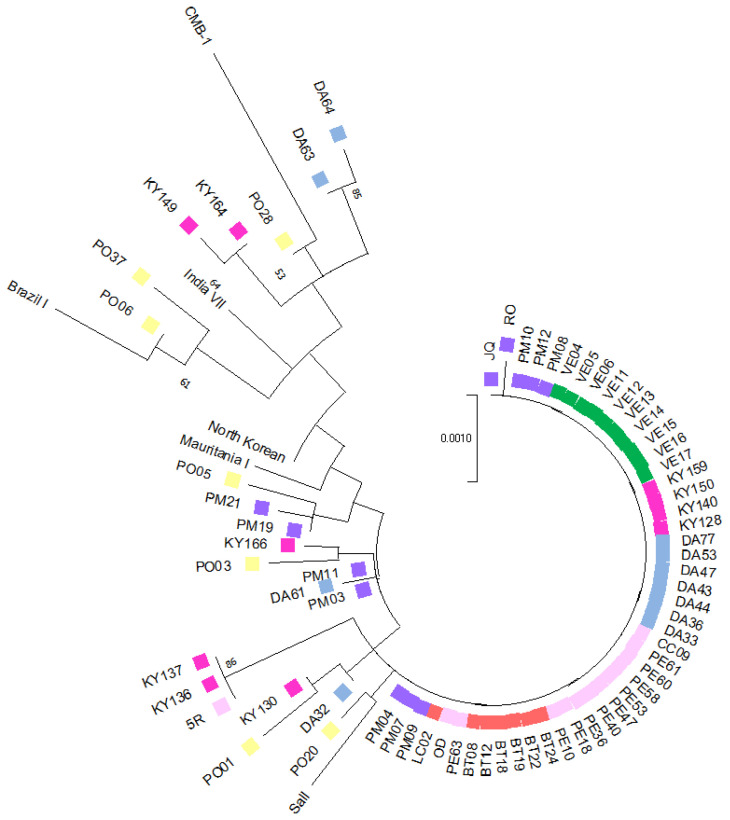
Phylogenetic tree constructed from concatenated sequences of four potential drug resistance markers (*pvcrt*-o, *pvmdr1*, *pvdhfr*, and *pvdhps*) from 63 *Plasmodium vivax* indigenous isolates, including 7 from Bocas del Toro (red color), 10 from Veraguas (green color), 12 from the Metropolitan area (violet color), 13 from Panama Este (pink color), 10 from Guna Yala (fuchsia color), and 11 from Darién (light blue color), and 7 imported isolates (yellow color), including 3 from Venezuela, 1 from Peru, 1 from Brazil, 1 from India, and 1 from China. For comparative purposes, six reference isolates from malaria-endemic regions worldwide, retrieved from the GenBank database, were also included. The phylogenetic tree was estimated by the maximum likelihood method with the Jukes–Cantor model. The final analysis incorporated 76 nucleotide sequences, covering a total of 2738 positions in the final dataset.

Phylogenetic tree reconstruction and a haplotype network analysis were conducted using concatenated sequences of four potential drug resistance markers (*pvcrt*-o, *pvmdr1*, *pvdhfr*, and *pvdhps*) from 63 *P. vivax* indigenous isolates (7 from Bocas del Toro, 10 from Veraguas, 12 from the Metropolitan area, 13 from Panama Este, 10 from Guna Yala, and 11 from Darién) and 7 imported isolates (3 from Venezuela, 1 from Peru, 1 from Brazil, 1 from India, and 1 from China). For comparative purposes, six reference isolates from malaria-endemic regions worldwide, retrieved from the GenBank database, were also included. The phylogenetic tree was estimated by the maximum likelihood method with the Jukes–Cantor model. The final analysis incorporated 76 nucleotide sequences, covering a total of 2738 positions in the final dataset (Figure 3). The complete sequence alignment of the concatenated genes from this study is available upon request.

A significant proportion of the local samples (76.2%; 48/63) were genetically homogeneous, forming a predominant, well-defined clade that encompassed all samples from Western Panama (17/17), while 56% (23/41) of samples were from Eastern Panama, and 67% (8/12) were from the Panama Metro region. Notably, none of the imported cases clustered within this clade. Samples from Eastern Panama exhibited greater genetic diversity, clustering into distinct subclades and showing higher genetic relatedness to imported cases and reference sequences from various geographical regions. Three local samples from Eastern Panama (two from Guna Yala and one from Panama Este) were grouped separately in a distinct clade. The phylogenetic analysis also allowed inferences to be made regarding the geographic origin of four samples from the Panama Metro region with unclear travel histories. Two of these samples (PM03 and PM11) clustered with a sample from Darién (DA61), while the other two were more genetically related to an imported sample from Iquitos, Peru. Imported samples were distributed across different subclades, clustering with reference sequences from diverse global regions. Interestingly, one imported sample from China exhibited close genetic similarity with samples from Guna Yala (KY130) and Darién (DA32), both located in Eastern Panama. Regarding imported cases, a sample from India (PO06) clustered within the same clade as a sample from Venezuela (PO37) and a reference strain from Brazil. The remaining imported samples were distributed across separate clades, not always grouping with reference strains from their reported geographic origins.

**Figure 4:**
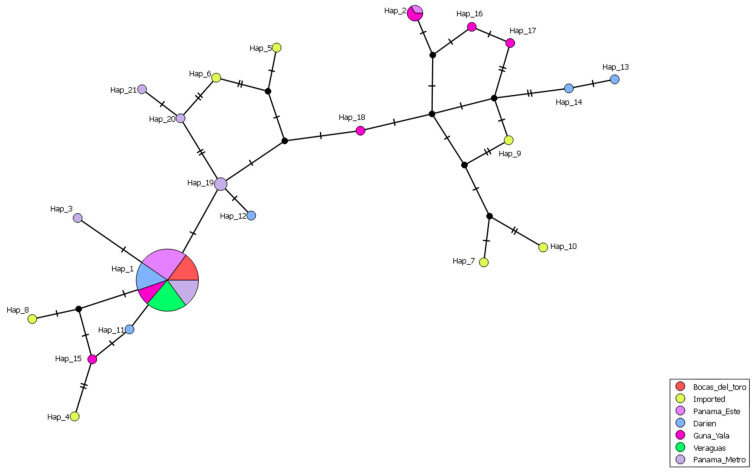
A haplotype network inferred by a median-joining method using concatenated sequences of four potential drug resistance markers (*pvcrt*-o, *pvmdr1*, *pvdhfr*, and *pvdhps*) from 63 *Plasmodium vivax* indigenous isolates, including 7 from Bocas del Toro (red color), 10 from Veraguas (green color), 12 from the Metropolitan area (violet color), 13 from Panama Este (pink color), 10 from Guna Yala (fuchsia color), 11 from Darién (light blue color), and 7 imported isolates (yellow color), including 3 from Venezuela, 1 from Peru, 1 from Brazil, 1 from India, and 1 from China. The circles represent an independent sequence haplotype with the color denoting the geographic origin and the size of the circle accounting for its frequency. The length of lines connecting haplotypes is proportional to the relatedness distance. A higher number of hatch marks represents greater genetic divergence between connected haplotypes.

The haplotype network analysis, based on the concatenated gene sequences (70 samples from this study and seven reference isolates from malaria-endemic countries), revealed the presence of 21 distinct haplotypes (Figure 4). Haplotype 1 was the predominant and most widespread haplotype, present in autochthonous samples across endemic regions in both Western and Eastern Panama (75%; 47/63). However, this haplotype was absent in imported cases. Notably, haplotype 1 was the only haplotype identified in Western Panama, whereas in Eastern Panama, 14 additional haplotypes were observed in autochthonous cases. Among these, only haplotype 2 contained more than one sample, comprising two from Guna Yala and one from Panama Este. The remaining haplotypes from autochthonous samples were each represented by a single sample.

Four distinct haplotypes (H3, H19, H20, and H21) were identified as singletons from the Panama Metro region, where their precise geographic origins were unclear. Of these, haplotype 3 exhibited close genetic relatedness to the predominant autochthonous haplotype 1, likely indicating that it evolved from the latter. Haplotype 19 was closely related to a haplotype from eastern Darien, while haplotypes 20 and 21 were genetically related to haplotypes composed of imported cases from Perú (H6-P005). In the case of imported samples, seven different haplotypes were identified, each corresponding to a single sample.

### 3.3. Genetic Diversity Results

The genetic diversity of *P. vivax* was assessed in isolates from two malaria-endemic regions in Panama (Eastern and Western regions) and imported cases (Table 1). Significant differences were observed among the groups. Imported cases exhibited the highest genetic diversity, with 13 segregating sites, seven haplotypes, and a haplotype diversity of 1.0. Nucleotide diversity (π = 0.00193) and 14 mutations were detected, reflecting the heterogeneous origins of these isolates. Eastern Panama showed moderate genetic diversity, with 13 segregating sites and 14 haplotypes (Hd = 0.576; π = 0.00083). The negative Fu’s Fs statistic (−5.450) suggested a recent population expansion or selective pressure. In contrast, Western Panama demonstrated no genetic diversity, with all isolates being genetically identical (Hd = 0; π = 0). This homogeneity indicates a clonal population structure, potentially due to limited gene flow or ecological constraints.

## 4. Discussion

Panama is a low-endemic malaria country located at the southernmost point of Mesoamerica, bordering Colombia to the southeast and Costa Rica to the north (Figure 1). Due to its strategic geographic position connecting South and North America, Panama is an excellent region to monitor and predict the potential emergence and spread of drug-resistant malaria parasites in Mesoamerica. Here, we conducted a retrospective analysis of *P. vivax* samples to detect mutations potentially associated with drug resistance from two geographically and ecologically separate malaria-endemic regions in Panama located on both sides of the Panama Canal, and from imported cases assumed on the travel history declared by the patient.

In recent decades, malaria transmission in Panama has been primarily confined to communities within Amerindian reservations situated in the eastern and western regions of the country (Figure 1). The eastern endemic region, in contrast to the west, has been exposed to distinct epidemiological risk factors that have contributed to malaria transmission, including greater vector diversity and a rising influx of irregular migrants entering through the porous border between Panama and Colombia [25]. This scenario has impacted the local epidemiology, with a notable increase in imported malaria cases and a greater genetic diversity observed in circulating *P. vivax* and *P. falciparum* parasites in the eastern region [26,27]. Interestingly, the central region of Panama has remained free of autochthonous malaria for decades, while in the western region, *P. vivax* transmission has been the only species recorded since the early 1960s [7].

Two genes (*pvcrt*-o and *pvmdr1*) have been proposed as candidates to monitor *P. vivax* resistance to chloroquine. None of the samples analyzed in this study, local or imported, harbored mutations in the coding region of *pvcrt*-o related to CQ resistance. Additionally, the *pvmdr1* Y976F mutation, also associated with reduced susceptibility to CQ, was detected in only one imported case and in two samples with unclear origin (Table 1 and Table S2). This mutation was previously described in three suspected imported cases isolated between 2005 and 2017 from a study conducted in Honduras [28].

Mutations in the *pvdhfr* gene associated with pyrimethamine resistance (S58R and S117N) were observed in most imported samples (6/7; 86%) and in some local samples from Eastern Panama near the Colombian border (8/44; 18%), suggesting possible selective pressures related to the use of antifolate drugs in this region of the country. Although antifolates are not recommended to treat *P. vivax* infections, the parasite can still experience selection pressure, especially in regions near the Colombian border where *P. vivax* and *P. falciparum* coinfections can occur. In fact, to contain the spread of malaria during the 2004–2006 period, sulfadoxine-pyrimethamine was extensively used in Eastern Panama for many years as a first-line treatment for uncomplicated *P. falciparum* cases [29].

Limited polymorphism was observed in the number of tandem repeats in the *pvdhfr* gene. All local and imported samples, except one, harbored the type C variant, which is also frequently observed in *P. vivax* isolates from Southeast Asia and South America [16,30,31,32]. One suspected imported sample harbored allelic variant type B. This sample exhibited a distinct genetic profile, including the Y976F-F1076L nonsynonymous mutations in the *pvmdr1* gene and a 12 bp deletion in the *pvdhps* gene, further supporting its possible imported origin. The type B variant has previously been reported in isolates from Mesoamerica (Honduras, Nicaragua, and México), as well as from South America and Southeast Asia [16,30,31,32]. While this variation has not been clearly associated with antifolate resistance [33], it can be used to assess genetic diversity and to track transmission and migration patterns. Mutations in the *pvdhps* gene associated with sulfadoxine resistance were only detected in a suspected imported case. Similarly, previous studies from Mesoamerican countries (Southern Mexico, Nicaragua, and Honduras) did not identify antifolate resistance-related mutations in local *P. vivax* populations [28,31].

Although drug resistance candidate genes may not be ideal for conducting genetic diversity studies due to the strong selective pressures to which they are subjected, our results evidence a distinct population structure, where parasites from Western Panama were completely homogeneous, in contrast to the significantly more diverse eastern populations. This diversity likely reflects higher transmission intensity and increased genetic influx in the eastern side, potentially driven by imported cases. It also might be influenced by geographical and ecological differences that characterize each region. A similar diversity pattern was observed when analyzing autochthonous *P. vivax* isolates in Panama [27,34]. As expected, due to their different geographical origin, a high diversity among imported parasites was observed.

Our study has several important limitations. First, the study sample size was relatively small and with a limited timeframe, spanning from 2004 to 2020. Consequently, our findings may not necessarily capture the entire or current genetic diversity of *P. vivax* in the country. Nevertheless, our results provide a valuable baseline for planning future molecular surveillance studies to detect the emergence and monitor the spread of drug-resistant *P. vivax* in the region. Second, only four *Plasmodium* genes under selective pressure were sequenced to evaluate the resistance profile and genetic diversity of the parasites. An analysis of additional and highly polymorphic genes would likely provide a better resolution and more robust insights into population dynamics. To address this, we implemented a multilocus sequence typing approach (MLST) by combining and analyzing four loci together. This approach provided insights into the genetic differentiation between populations from Western and Eastern Panama and identified unique genetic profiles in imported cases. A recent study conducted in Honduras, Mesoamerica, also employed an MLST approach by combining four *Pvmsp3α* and *Pvmsp3β* loci, and it demonstrated to be more effective in assessing the genetic diversity of *P. vivax* compared to individual markers [35]. Nevertheless, MLST has potential limitations related to the number and selection of genes to be included in the analysis, as well as bias inferences when dealing with high levels of heterozygosity.

Third, travel history might not be reliable to determine the true geographic origin of infection. This is particularly important for tracking imported malaria cases in Mesoamerica, where extracontinental migrants are frequent and may acquire malaria infection along their northward migration routes that include several South American countries [13]. For example, in our study, an imported sample from China, based on the declared travel history, was genetically more related to local samples from Eastern Panama, while a sample declared as being imported from India shared closer genetic relationships with samples from Venezuela and Brazil (Figure 3).

While limitations related to MLST and the true origin of infections can be more precisely inferred with advanced whole genome and next-generation sequencing techniques, the high cost of the MLST approach remains a significant limitation for developing countries in Mesoamerica, where malaria is endemic but highly focalized, and funding can instead be prioritized in actionable field measures, like mass drug administration [36,37] informed by molecular surveillance, like the method we described here, in order to reduce transmission and achieve elimination.

## 5. Conclusions

No chloroquine resistance-associated mutations were detected in autochthonous *P. vivax* isolates from Panama, a molecular finding in agreement with reports of the high clinical efficacy of chloroquine in the country. Similarly, the prevalence of mutations potentially associated with resistance to antifolates was very low in local isolates. The dhfr S117N mutation was detected in seven samples, and the dhfr S58R mutation was detected in two isolates. All of these mutations were detected in samples from eastern communities near the Colombian border, a region with significant cross-border migration and potential drug selection pressures. Overall, the genetic diversity of local *P. vivax* populations in Panama was low. However, significant differences were observed between the eastern and western regions of the country (Table 1). Isolates from the western side were completely homogenous, suggesting a clonal expansion. In contrast, eastern isolates exhibited higher genetic diversity, consistent with the region’s different epidemiological characteristics, which includes, among others, higher transmission rates, the coexistence of *P. falciparum*, higher vector species diversity, and the frequent introduction of imported cases. These findings underscore the need to continue molecular surveillance to inform *P. vivax* control strategies, especially those related to drug use, both in Panama and across all of Mesoamerica.

## Figures and Tables

**Table 1:** Comparison of drug resistance (*pvcrt*-o, *pvmdr1*, *pvdhfr*, and *pvdhps*) gene diversity among *Plasmodium vivax* isolates from two separate geographical and ecological malaria-endemic regions in Panama and imported cases.

Region	N	Sites	S	H	Hd	π	Eta	k	Fu’s Fs Statistic
Imported	7	2720	13	7	1	0.00193	14	5	−2898
Eastern Panama	46	2738	13	14	0.576	0.00083	13	2	−5450
Western Panama	17	2720	0	1	0	0	0	0	0

N = number of analyzed sequences; S = number of segregating sites; H = number of haplotypes; Hd = haplotype diversity sequences; π = nucleotide diversity; Eta = total number of mutations; k = average number of nucleotide differences.

## Data Availability

All data underlying the results from this study are provided as part of the article in tables and figures. DNA sequences were deposited in GenBank as described in the methodology.

## Supplementary Materials

The following supporting information can be downloaded at https://www.mdpi.com/article/10.3390/pathogens14030231/s1, Table S1: GenBank accession numbers for *Plasmodium vivax* drug resistance genes analyzed in this study; Table S2: Mutations in potential drug resistance genes in *Plasmodium vivax* isolates from malaria-endemic regions in Panama and imported cases.

## Abbreviations

The following abbreviations are used in this manuscript:

CQ	Chloroquine
ICGES	Instituto Conmemorativo Gorgas de Estudios de la Salud
MLST	Multilocus sequence typing approach
LD	Linear dichroism
NMEP	National Malaria Elimination Plan
PQ	Primaquine
ssrRNA	Small subunit ribosomal RNA
WHO	World Health Organization
